# A Rare Case of Bilobed Gastric Duplication Cyst in a Four-Month-Old Infant

**DOI:** 10.7759/cureus.81435

**Published:** 2025-03-29

**Authors:** Yacine Zouirech, Abir Manni, Bashar Al Jabary, Jaouad Bouljrouf, Mounir Kisra

**Affiliations:** 1 Pediatric Surgery Department "A", Ibn Sina University Hospital Center, Children's Hospital, Rabat, MAR; 2 Faculty of Medicine and Pharmacy, Mohammed V University, Rabat, MAR

**Keywords:** abdominal cystic mass, bilobed cyst, congenital anomalies, gastric duplication cyst, non-bilious vomiting, pancreatic heterotopia, pediatric surgery

## Abstract

Gastric duplication cysts (GDCs) are rare congenital anomalies of the gastrointestinal tract that can present with nonspecific symptoms, leading to diagnostic challenges and potential complications. We report the case of a four-month-old female infant who presented with persistent nonbilious vomiting, failure to thrive, and abdominal distension. Imaging (abdominal ultrasound and CT scan) revealed a bilobed intra-peritoneal cystic mass, consistent with a GDC. The patient underwent successful surgical resection via laparotomy, including partial resection of the transverse colon. Mucosal cauterization was employed to preserve gastric integrity and minimize extensive resection. Histopathological analysis confirmed a GDC with pancreatic heterotopia. The postoperative course was uneventful, with complete symptom resolution and normal growth. This case underscores the significance of early diagnosis, appropriate imaging, and precise surgical intervention to prevent complications and optimize patient outcomes.

## Introduction

Alimentary tract duplications are rare congenital anomalies that can occur anywhere along the digestive tract, from the oral cavity to the anus, with an estimated incidence of one in 4,500 live births [1]. Among these, the ileum is the most commonly affected site, followed by the esophagus, jejunum, colon, stomach, and appendix [1]. Gastric duplications (GDs) are particularly uncommon, representing only 2-9% of alimentary tract duplications, with an estimated incidence of 1.7 per 1,000,000 live births [1,2].

GDs are characterized by cystic or tubular structures that share a common wall with the stomach but possess distinct mucosal linings [3]. Most are located along the greater curvature of the stomach [1,2,4]. Two forms of GDs have been described: the cystic type, which accounts for approximately 80% of cases and is typically non-communicating with the gastric lumen, and the tubular type, which is less common and often communicates with the lumen [1].

GDs are most frequently diagnosed in early childhood, often within the first year of life [2,4]. Their clinical manifestations vary depending on the age of the patient, size, location, and type of the lesion [1,2,4]. Common symptoms include vomiting, abdominal pain, weight loss, and a palpable abdominal mass. However, some cases may remain asymptomatic and are detected incidentally during prenatal imaging or routine physical examinations [1,2,4,5].

Advanced imaging techniques, such as ultrasound, CT, and MRI, play a crucial role in diagnosis. These modalities often reveal cystic lesions that may cause tissue compression, obstruction, or vomiting [4,6]. Nevertheless, due to nonspecific clinical and imaging findings, an accurate diagnosis can be challenging, frequently requiring surgical exploration and histopathological confirmation [1,2,4].

Given the potential complications of GDs, including hemorrhage, perforation, obstruction, and malignant transformation, early surgical intervention is strongly recommended. Surgical resection remains the gold standard treatment, offering favorable outcomes [1,2,4,7,8].

Due to the rarity of reported cases and the variability in their presentation and management, documenting each occurrence is essential. Such reports contribute to a deeper understanding of this condition and help refine diagnostic and therapeutic strategies.

## Case presentation

A four-month-old female infant, born to consanguineous parents (first-degree relatives) following an unmonitored pregnancy and non-medicalized vaginal delivery (birth weight: 2.5 kg), presented with persistent nonbilious vomiting since birth (one to two episodes/day, 30-40 mL/episode, occurring 10-15 minutes post-feeding). Initially managed as gastroesophageal reflux disease (GERD), her symptoms worsened significantly over the preceding seven days, with increased vomiting frequency (three to six episodes/day), refusal to feed, and progressive abdominal distension. This exacerbation occurred in the context of fever (39°C), prompting evaluation by a general practitioner and pediatrician. Symptomatic treatment provided only partial relief. The family history was unremarkable for similar gastrointestinal disorders.

On admission, the infant exhibited a failure to thrive (weight: 4.6 kg, <5th percentile for age). Abdominal palpation revealed a soft, fluctuant, and non-tender mass in the left hypochondrium, accompanied by mild distension and no guarding. The systemic examination was otherwise unremarkable.

Diagnostic workup

Laboratory investigations revealed hypochromic microcytic anemia and mild electrolyte imbalances. Serology for Epstein-Barr virus (EBV), cytomegalovirus (CMV), and hepatitis A/B/C was negative. Cerebrospinal fluid analysis ruled out infectious meningitis.

**Figure 1:**
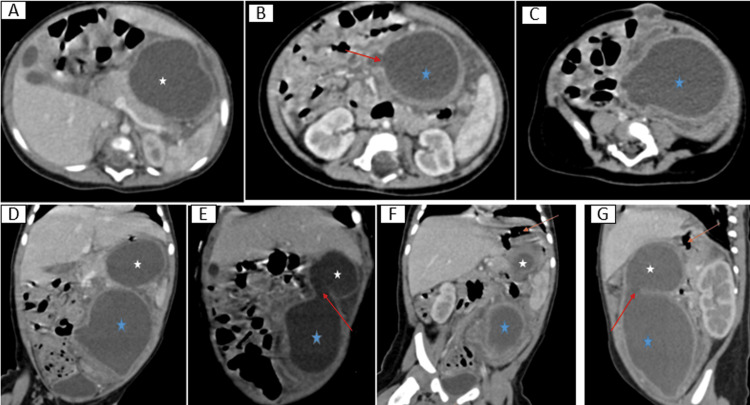
Contrast-enhanced CT images in axial (A, B, C), coronal (D, E, F), and sagittal (G) views of a four-month-old infant with a bilobed GDC. The images demonstrate a bilobed intraperitoneal cystic mass extending from the left hypochondrium to the left iliac fossa, measuring 51 mm × 47 mm × 117 mm (D, E, G). The bilobed structure exhibits clear communication between the two lobes (E, F) but no connection with the digestive lumen. White star (★): primary GDC. Blue star (★): intra-epiploic extension of the GDC. Red arrows (→): communication between the two lobes (B, E, G). Orange arrows (→): areas of close adhesion to the gastric wall (F, G). GDC, gastric duplication cyst

Abdominal ultrasound identified two hypoechoic cystic formations in the left hypochondrium, measuring approximately 40 mm × 30 mm and 37 mm × 27 mm. Further contrast-enhanced CT confirmed a bilobed intra-peritoneal cystic mass, extending from the left hypochondrium to the left iliac fossa, with dimensions of 51 mm × 47 mm × 117 mm. The mass demonstrated clear communication between the two lobes but had no connection to the digestive lumen. The cyst walls were thick, regular, and morphologically similar to the gastric wall, with firm adhesion to both the gastric wall and intestinal mesentery, strongly suggesting a diagnosis of gastric duplication cyst (GDC) (Figure 1).

Surgical intervention

**Figure 2:**
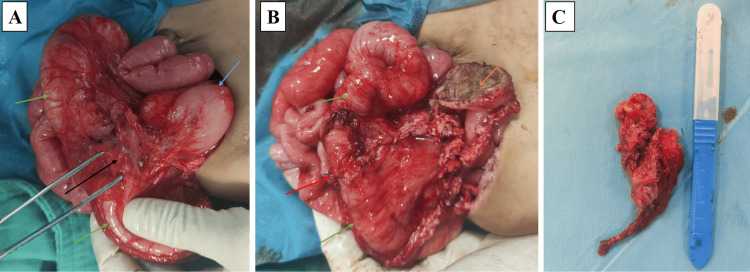
Intraoperative images illustrating the resection of a bilobed GDC. (A): The primary GDC, originating from the gastric antrum (blue arrow), communicates with its intra-epiploic extension (black arrow), which infiltrates the transverse colon (green arrow), consistent with the characteristics of a bilobed GDC. (B): Digestive continuity was successfully restored via a terminal anastomosis (red arrow). Mucosal electrocoagulation (orange arrow) was applied to manage the gastric attachment, effectively preventing the need for gastric resection. (C): The excised bilobed cystic specimen, illustrating its characteristic size and morphology. GDC, gastric duplication cyst

Given these findings, surgical intervention was deemed necessary. An exploratory laparotomy was performed through an upper abdominal horizontal incision, revealing dense adhesions between the transverse colon and stomach. Careful dissection identified a cystic mass on the gastric antrum, without communication with the gastric lumen, a hallmark feature of GDCs. The intra-epiploic extension of the cyst infiltrated the transverse colon and adhered closely to the tail of the pancreas and splenic hilum, confirming the diagnosis of a bilobed GDC (Figure 2A).

To facilitate removal, the anterior cyst wall was excised, revealing clear yellow serous fluid within the cyst. The posterior cystic wall appeared to share a muscular layer with the stomach, but no luminal connection to the stomach was present. The mucosal layer was resected, preserving the muscular layer of the posterior wall. Following mucosal excision, cauterization with an electric scalpel was performed to eradicate any residual mucosal surface, avoiding the need for gastric resection. The intra-epiploic extension of the cyst was meticulously dissected and removed, requiring resection of a 26 mm segment of the infiltrated transverse colon. Digestive continuity was restored via terminal anastomosis (Figure 2B).

Pathological findings

**Figure 3:**
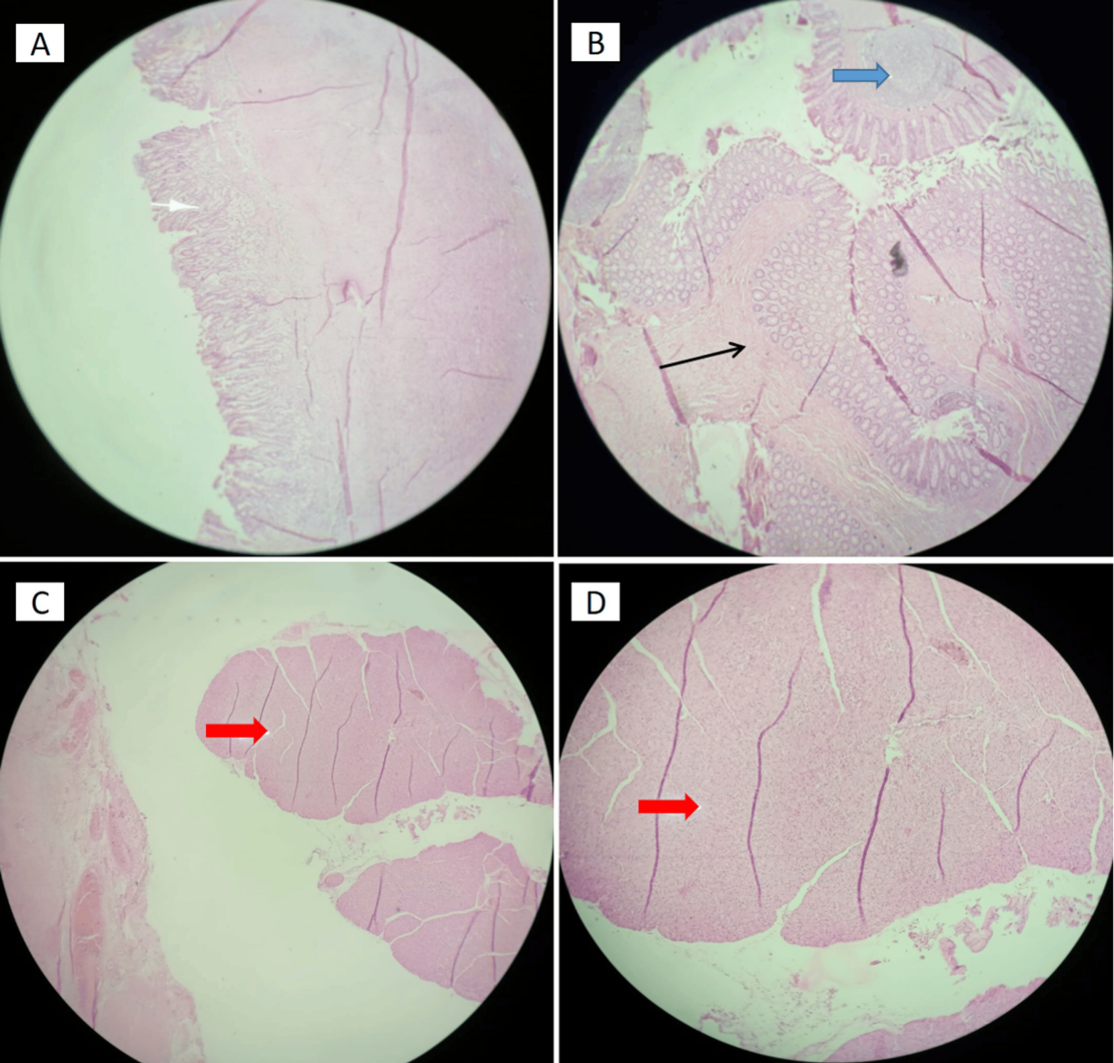
Histological examination of the bilobed GDC (H&E staining). (A): Gastric wall lined with antral-type gastric mucosa, showing well-organized epithelial structures and a thick muscular layer (white arrow; H&E ×20). (B): Colonic mucosa with well-differentiated glands and an organized muscular layer (black arrow), along with lymphoid follicles (blue arrow; H&E ×20). (C) and (D): Heterotopic pancreatic exocrine tissue (red arrow) in the perigastric region (H&E ×20 and ×40, respectively). GDC, gastric duplication cyst

Macroscopic examination revealed two fragments. The first fragment (5 cm × 4 cm) corresponded to the primary GDC, connected to a 3.5 cm × 1 cm intra-epiploic extension, while the second fragment (2.6 cm × 1.2 cm) represented the resected transverse colon (Figure 2C). Microscopic examination confirmed the diagnosis. The first fragment exhibited antral-type gastric mucosa with a thick muscular layer, characteristic of GDCs (Figure 3A). The intra-epiploic cystic extension showed ulcerated mucosa at the interface between the gastric and colonic components. The second fragment revealed colonic mucosa with well-differentiated glands, an organized muscular layer, and lymphoid follicles, corresponding to the resected transverse colon (Figure 3B). Heterotopic pancreatic exocrine tissue was identified in the perigastric region, a rare but recognized feature of GDCs (Figure 3C, 3D). No histological evidence of malignancy was observed, confirming the benign nature of the lesion.

Postoperative course and follow-up

Following a four-day postoperative fasting period, the patient resumed oral ingestion of clear liquids. As the patient remained asymptomatic, no contrast study was performed. In the absence of complications after reintroducing oral intake, the patient was discharged on the seventh postoperative day.

During follow-up visits at one, three, six, and 12 months postoperatively, the patient exhibited no gastrointestinal symptoms, no recurrence of cysts on ultrasonography, and continued to thrive. At the 12-month follow-up, her weight was 9.1 kg (25th percentile), confirming the resolution of failure to thrive.

## Discussion

Overview and pathogenesis

GDCs are rare congenital anomalies, accounting for only 3-5% of all digestive tract duplications [4]. While some studies suggest a higher prevalence in females, others report a greater occurrence in males [1,2,4]. However, systematic reviews by Li Y et al. (2021) and Aodhnait S. Fahy (2019) found no significant gender-based differences in their distribution [1,9]. Our case describes a bilobed GDC with an intra-epiploic extension, a highly uncommon anatomical presentation. Although most GDCs are solitary, cases of multiple duplications have been documented. For instance, Liu F et al. reported two cases of multiple GDCs among 17 children, while Luo Y et al. described one case involving three GDCs at different locations among 31 cases [2,4]. The “dumbbell-like” morphology observed in our case has been previously reported only once by Kumar, further emphasizing its exceptional rarity [10].

The exact pathogenesis of GDCs remains unclear and is thought to involve aberrations during embryogenesis between the fourth and eighth weeks of gestation. One hypothesis suggests that the detachment of a group of cells during the differentiation of the foregut into the stomach leads to the formation of a duplicate structure [4,8]. Several theories have been proposed to explain GDC development, though none fully account for their formation. The widely accepted split notochord theory posits that incomplete separation of the notochord from the endoderm results in a duplicated gastrointestinal segment [1]. Another proposed mechanism, the incomplete twinning hypothesis, suggests that partial duplication of the gastrointestinal tract during embryonic development leads to a secondary gastric structure [8]. Other theories include McLetchie’s theory, phylogenetic reversion, aberrant luminal canalization, and intrauterine trauma [1,2,4]. While each theory provides valuable insights, the pathogenesis of GDCs is likely multifactorial [1].

Clinical presentation and diagnosis

Most GDCs are diagnosed prenatally through routine fetal ultrasound or during physical examinations in newborns and children. However, some cases remain asymptomatic and are incidentally discovered during imaging for unrelated conditions [1,2,4,6]. The clinical presentation of GDCs is highly variable, ranging from gastric outlet obstruction to completely asymptomatic cases [2,4,10]. Symptoms largely depend on the size, location, and type of the cyst, as well as the patient's age at onset, which frequently contributes to delayed diagnosis [1-4]. Initially, the cyst is small and asymptomatic, but as it grows, the secretion of gastric juice increases, leading to elevated tension and the onset of clinical symptoms [4].

In pediatric patients, GDCs commonly present with vomiting, abdominal pain, melena, abdominal distension, gastric outlet obstruction, and failure to thrive [1,2,4]. Physical examination may reveal abdominal tenderness or a palpable mass in the upper abdomen [1,5]. In older children, abdominal pain, weight loss, and distension are more frequently observed, whereas vomiting and gastrointestinal hemorrhage are the predominant symptoms in infants [1,2]. Acute complications such as infection, bleeding, or perforation may lead to an emergent presentation [11].

Imaging and diagnostic tools

Imaging studies are fundamental in the diagnosis and management of GDCs, providing crucial anatomical details for surgical planning. Ultrasound is the preferred initial imaging technique due to its non-invasive nature, affordability, and accessibility. It allows for a precise assessment of the cyst’s size, wall thickness, and vascular supply [1,2,6]. A characteristic "double wall sign" (a hyperechoic inner layer and a hypoechoic outer layer) is often observed and serves as a key diagnostic feature of GDCs [4,9,12]. However, ultrasound has limitations, particularly in cases complicated by gastrointestinal gas, bleeding, or infection, often necessitating further evaluation with CT or MRI [4,9]. Prenatal diagnosis of GDCs is increasingly feasible, facilitating early postnatal management. In a study by Liu F et al., eight out of 17 cases (47%) were diagnosed via ultrasound, with seven detected prenatally and one postnatally [2,4].

CT scans are superior to ultrasound in defining the precise location and relationships of the cyst with adjacent structures, making them valuable tools for surgical planning [2,6,12]. MRI is particularly useful for differentiating GDCs from other cystic lesions, such as pancreatic pseudocysts or mesenteric cysts, by providing high-resolution images of internal structures and their associations with the gastric wall [8,12].

Although plain abdominal radiography is not routinely used for GDC diagnosis, it may provide indirect signs, such as soft-tissue interposition between the gastric shadow and the transverse colon [10]. Upper gastrointestinal radiography, selectively employed for large or luminally connected cysts, may reveal filling defects or a niche shadow within the gastric wall, suggestive of a GDC [2].

Digestive endoscopy is a key diagnostic tool in the preoperative assessment of GDCs. It allows direct visualization of the lesion, aiding in classification and precise localization while ruling out differential diagnoses, such as gastric ulcers or diverticula [4,2,11].

The definitive diagnosis of GDCs is established through surgical exploration and histopathological examination, with criteria set by Rowling in 1959. According to Rowling, GDCs must have walls contiguous with the stomach, smooth muscle continuous with the gastric wall, and a lining of alimentary epithelium, which may include gastric, colonic, or jejunal mucosa or other gastrointestinal mucosa [3].

Differential diagnosis and associated anomalies

Unlike diverticula, GDCs typically share both a common wall and blood supply with the stomach [1]. Moreover, GDCs are distinguished by an epithelial lining that may differ from the surrounding gastrointestinal tissue, with their classification often based on the site of attachment [7]. Histologically, ectopic mucosa is a significant finding, with gastric mucosa present in 20-30% of gastrointestinal duplication cysts (GIDCs) and ectopic pancreatic tissue being the most frequently associated anomaly with GDs [5].

Accurate preoperative diagnosis of GDCs is crucial, as they can be mistaken for various conditions depending on their location, nature, and type. Differential diagnoses include other cystic masses in the upper abdomen, such as pancreatic cysts, adrenal cysts, intestinal duplications, mesenteric cysts, neuroblastomas, and lymphangiomas [8,12]. Additionally, GDCs may mimic gastric-specific conditions, such as diverticula, ulcers, or tumors, including rare cases like gastric adenomyomas misdiagnosed as GDCs [4,2,11]. Ensuring accurate differentiation is critical to avoid missed or incorrect diagnoses and to facilitate appropriate treatment planning.

Surgical management and prognosis

Surgical resection remains the definitive treatment for GDCs, with most studies advocating for resection even in asymptomatic cases once the diagnosis is confirmed. This recommendation stems from the significant risks of complications such as bleeding, perforation, infection, obstruction, and malignant transformation [1,4,2,8]. Malignant transformation in GDCs should be suspected if solid components are identified or if serum CEA/CA199 levels are elevated [1,10]. Unfortunately, even with radical surgery, the prognosis for GDCs with malignancy is generally poor, emphasizing the necessity of prompt surgical intervention upon diagnosis [1,2,4].

The choice of surgical technique, from laparotomy to laparoscopy or endoscopy-assisted laparoscopic surgery (EALS), depends on factors such as the cyst's size, location, accessibility, relationship with surrounding structures, and the expertise of the surgical team [1,2,4]. Minimally invasive approaches like laparoscopy and EALS have demonstrated advantages, including shorter surgical times, reduced complications, and enhanced precision in cyst removal, making them valuable options in selected cases [4].

Extra-mucosal excision, with preservation of the gastric wall, is the preferred surgical approach for managing GDCs. However, partial gastric resection may be required if the cyst shares a common wall or blood supply with the stomach [1,4,2]. For cases where cysts closely adhere to vital structures or present challenges in surgical exposure, advanced techniques such as mucosectomy or mucosal electrocoagulation can be utilized to ensure effective management [2]. In this case, laparotomy was chosen due to the significant size of the cyst and its infiltration into the transverse colon. Complete resection, including a segment of the colon, was successfully performed, and digestive continuity was restored through terminal anastomosis. Mucosal electrocoagulation proved effective in managing the gastric attachment, eliminating the need for gastric resection.

Postoperative care and outcomes

Postoperative care for GDCs is typically uncomplicated, with early feeding initiated through nasogastric or nasointestinal tubes within the first one to five days, followed by a gradual transition to oral feeding [2,4]. Vigilant monitoring is crucial to identify potential complications such as bleeding, infection, anastomotic leakage, fistulas, or intestinal obstructions [4,2]. Recurrence of symptoms is rare after complete cyst removal [2,4]. In the present case, oral feeding was successfully resumed on postoperative day 4, and the infant showed no recurrence of symptoms during follow-up, emphasizing the significance of prompt diagnosis and thorough surgical management. The prognosis following complete surgical resection is excellent, with most patients achieving full recovery and remaining symptom-free [1,2,4].

## Conclusions

GDCs are rare congenital anomalies that pose significant diagnostic challenges due to their nonspecific symptoms, often leading to delayed diagnosis and increased risk of complications such as obstruction, infection, and, in rare cases, malignancy. This case highlights the importance of considering GDCs in infants presenting with persistent nonbilious vomiting, where early recognition and intervention are critical to preventing long-term morbidity. Advanced imaging modalities, including contrast-enhanced CT and ultrasound, are instrumental in characterizing cyst features and guiding surgical planning. Tailored surgical approaches, such as extra-mucosal excision or mucosal electrocoagulation, enable effective management while preserving gastric function.

The documentation and analysis of rare cases like this bilobed GDC contribute substantially to medical literature, offering insights into clinical presentations, diagnostic pathways, and optimal therapeutic strategies. Such reports are vital for refining diagnostic criteria, enhancing surgical techniques, and ultimately improving outcomes for pediatric patients with this uncommon condition. Continued sharing of clinical experiences will further advance the collective understanding and management of GDCs.
